# WSES consensus conference guidelines: monitoring and management of severe adult traumatic brain injury patients with polytrauma in the first 24 hours

**DOI:** 10.1186/s13017-019-0270-1

**Published:** 2019-11-29

**Authors:** Edoardo Picetti, Sandra Rossi, Fikri M. Abu-Zidan, Luca Ansaloni, Rocco Armonda, Gian Luca Baiocchi, Miklosh Bala, Zsolt J. Balogh, Maurizio Berardino, Walter L. Biffl, Pierre Bouzat, Andras Buki, Marco Ceresoli, Randall M. Chesnut, Osvaldo Chiara, Giuseppe Citerio, Federico Coccolini, Raul Coimbra, Salomone Di Saverio, Gustavo P. Fraga, Deepak Gupta, Raimund Helbok, Peter J. Hutchinson, Andrew W. Kirkpatrick, Takahiro Kinoshita, Yoram Kluger, Ari Leppaniemi, Andrew I. R. Maas, Ronald V. Maier, Francesco Minardi, Ernest E. Moore, John A. Myburgh, David O. Okonkwo, Yasuhiro Otomo, Sandro Rizoli, Andres M. Rubiano, Juan Sahuquillo, Massimo Sartelli, Thomas M. Scalea, Franco Servadei, Philip F. Stahel, Nino Stocchetti, Fabio S. Taccone, Tommaso Tonetti, George Velmahos, Dieter Weber, Fausto Catena

**Affiliations:** 1 Department of Anesthesia and Intensive Care, Parma University Hospital, Via Gramsci 14, 43100 Parma, Italy; 2 Department of Surgery, College of Medicine and Health Sciences, UAE University, Al-Ain, United Arab Emirates; 3 Department of General and Emergency Surgery, Bufalini Hospital, Cesena, Italy; 4 Department of Neurosurgery, Georgetown University School of Medicine, Washington, DC USA; 5 Department of Clinical and Experimental Sciences, University of Brescia, Brescia, Italy; 6 Trauma and Acute Care Surgery Unit, Hadassah Hebrew University Medical Center, Jerusalem, Israel; 7 Department of Traumatology, John Hunter Hospital, University of Newcastle, Newcastle, NSW Australia; 8 Department of Anesthesiology, CTO Hospital, Turin, Italy; 9 Division of Trauma and Acute Care Surgery, Scripps Memorial Hospital, La Jolla, CA USA; 10 Department of Anaesthesiology and Critical Care, Grenoble Alps Trauma Center, University Hospital of Grenoble-Alpes, Grenoble Cedex, France; 11 Department of Neurosurgery, Medical School, University of Pécs, Pécs, Hungary; 12 János Szentágothai Research Centre, University of Pécs, Pécs, Hungary; 13 Department of General and Emergency Surgery, ASST, San Gerardo Hospital, Monza, Italy; 14 School of Medicine and Surgery, University of Milan-Bicocca, Milan, Italy; 15 Department of Neurological Surgery, University of Washington, Harborview Medical Center, Seattle, WA USA; 16 General Surgery and Trauma Team, University of Milano, ASST Niguarda Milano, Milan, Italy; 17 Neuro-Intensive Care, Department of Emergency and Intensive Care, ASST, San Gerardo Hospital, Monza, Italy; 18 Riverside University Health System Medical Center, Loma Linda University School of Medicine, Moreno Valley, CA USA; 19 Colorectal Unit, Addenbrooke’s Hospital, Cambridge University Hospitals NHS Foundation Trust, Cambridge, UK; 20 Division of Trauma Surgery, Hospital de Clinicas, School of Medical Sciences, University of Campinas, Campinas, Brazil; 21 Department of Neurosurgery, All India Institute of Medical Sciences and associated Jai Prakash Narain Apex Trauma Centre, New Delhi, India; 22 Department of Neurology, Neurocritical Care Unit, Medical University of Innsbruck, Innsbruck, Austria; 23 Division of Neurosurgery, Department of Clinical Neurosciences, Addenbrooke’s Hospital and University of Cambridge, Cambridge Biomedical Campus, Cambridge, UK; 24 NIHR Global Health Research Group on Neurotrauma, University of Cambridge, Cambridge, UK; 25 Departments of General Acute Care, Abdominal Wall Reconstruction and Trauma Surgery, Foothills Medical Centre, Calgary, AB Canada; 26 Division of Trauma and Surgical Critical Care, Osaka General Medical Center, Osaka, Japan; 27 Department of General Surgery, Rambam Health Campus, Haifa, Israel; 28 Abdominal Center, Helsinki University Hospital Meilahti, Helsinki, Finland; 29 Department of Neurosurgery, Antwerp University Hospital and University of Antwerp, Edegem, Belgium; 30 Department of Surgery, Harborview Medical Centre, University of Washington School of Medicine, Seattle, WA USA; 31 Department of Trauma Surgery, Denver Health, Denver, CO USA; 32 Department of Intensive Care Medicine, St. George Clinical School, University of New South Wales and The George Institute for Global Health, Sydney, Australia; 33 Department of Neurosurgery, University of Pittsburgh Medical Center, Pittsburgh, PA USA; 34 Trauma and Acute Critical Care Center, Medical Hospital, Tokyo Medical and Dental University, Tokyo, Japan; 35 Department of Surgery, Trauma Surgery, Hamad General Hospital, Doha, Qatar; 36 INUB/MEDITECH Research Group, El Bosque University, Bogotá, Colombia; 37 MEDITECH Foundation, Clinical Research, Cali, Colombia; 38 Neurosurgery Department, Vall d’Hebron University Hospital, Universitat Autónoma de Barcelona, Barcelona, Spain; 39 General Surgery, Macerata Hospital, Macerata, Italy; 40 R Adams Cowley Shock Trauma Center, University of Maryland School of Medicine, Baltimore, MD USA; 41 Department of Neurosurgery, Humanitas University and Research Hospital, Milan, Italy; 42 College of Osteopathic Medicine, Rocky Vista University, Parker, CO USA; 43 Neuro ICU Fondazione IRCCS Cà Granda Ospedale Maggiore Policlinico, Milan, Italy; 44 Department of Physiopathology and Transplantation, Milan University, Milan, Italy; 45 Department of Intensive Care, Erasme Hospital, Université Libre de Bruxelles, Brussels, Belgium; 46 Division of Trauma, Emergency Surgery and Surgical Critical Care, Massachusetts General Hospital and Harvard Medical School, Boston, MA USA; 47 Trauma and General Surgery, Royal Perth Hospital, Perth, Australia; 48 Department of Emergency Surgery, Parma University Hospital, Parma, Italy

**Keywords:** Traumatic brain injury, Polytrauma, Bleeding, Hemorrhage, Monitoring, Management

## Abstract

The acute phase management of patients with severe traumatic brain injury (TBI) and polytrauma represents a major challenge. Guidelines for the care of these complex patients are lacking, and worldwide variability in clinical practice has been documented in recent studies. Consequently, the World Society of Emergency Surgery (WSES) decided to organize an international consensus conference regarding the monitoring and management of severe adult TBI polytrauma patients during the first 24 hours after injury. A modified Delphi approach was adopted, with an agreement cut-off of 70%. Forty experts in this field (emergency surgeons, neurosurgeons, and intensivists) participated in the online consensus process. Sixteen recommendations were generated, with the aim of promoting rational care in this difficult setting.

## Introduction

Traumatic brain injury (TBI), both isolated and in combination with extra-cranial lesions, is a global health problem associated with high mortality and disability [1, 2]. In addition, post-traumatic bleeding is a leading cause of preventable death among injured patients [3–5]. A multicenter observational study, involving 1536 trauma patients, identified exsanguination as the most frequent cause of early death [5]. The same study, however, found TBI as the most common cause of delayed mortality and disability [5]. Therefore, the combination of brain damage and extra-cranial injuries, causing bleeding, shock, and arterial hypotension, is especially challenging. On the one hand, bleeding can be rapidly life-threatening and has to be corrected promptly; in this regard, various strategies, often including “permissive arterial hypotension”, have been proposed [6–10]. On the other hand, arterial hypotension may exacerbate cerebral secondary damage and is associated with further worsening of the outcome [11].

A recent international survey revealed great variability in clinical practice during the acute phase management of polytrauma patients with TBI [12]. Moreover, guidelines regarding optimal monitoring and management strategies in this setting are lacking [10, 13]. Considering the above, the World Society of Emergency Surgery (WSES) promoted an international consensus conference on monitoring and management of severe adult TBI polytrauma patients during the first 24 hours after injury.

## Methods

A modified Delphi approach was adopted. Three subsequent online questionnaires were administered between January and May 2019. The agreed cut-off for the consensus was defined as 70% of experts in agreement, in keeping with recent initiatives in this field [14, 15]. Forty experts (emergency surgeons, neurosurgeons, and intensivists) in the management of severe TBI patients with polytrauma [Abbreviated Injury Score (AIS) ≥ 3 at least in 2 body regions] participated in the consensus process (see Appendix 1 in Additional file 1). Consensus statements were developed by 3 authors (EP, NS, and FC) based on a non-systematic literature search and evaluated by the expert panel through an electronic consultation. Sixteen recommendations related to monitoring and management of adult severe TBI patients with polytrauma in the acute phase (first 24 hours) were generated. Once a consensus (> 70% agreement) for each statement was achieved, a summary guideline, together with a corresponding algorithm, was circulated to all participants for the final acceptance. A summary of the data was presented and discussed at the 6th International WSES meeting held in Nijmegen (The Netherlands) from 26 to 28 June 2019. The present paper was drafted after the meeting and distributed to all participants for review and final approval before submission.

### Notes on the use of the current consensus

The aim of this consensus is to support clinician’s decision-making in the management of bleeding TBI polytrauma patients in the first 24 hours after injury. The included statements are created to assist the physician’s clinical judgment, which is necessary to provide appropriate (personalized) therapy. Advanced neuromonitoring and specific management strategies that can be indicated in a later stage are not addressed. Considering the lack of high-quality studies in this setting, we adopted a modified Delphi approach involving experts from different countries worldwide; this approach is probably less rigorous than evidence-based guidelines [13]. However, we think that our methodology can provide useful recommendations in this challenging clinical scenario.

The practice guidelines promulgated in this work do not represent a standard of practice. They are suggested plans of care, based on best available evidence and the consensus of experts, but they do not exclude other approaches as being within the standard of practice. However, responsibility for the results of treatment rests with those who are directly engaged therein, and not with the consensus group.

## Results

**Fig. 1:**
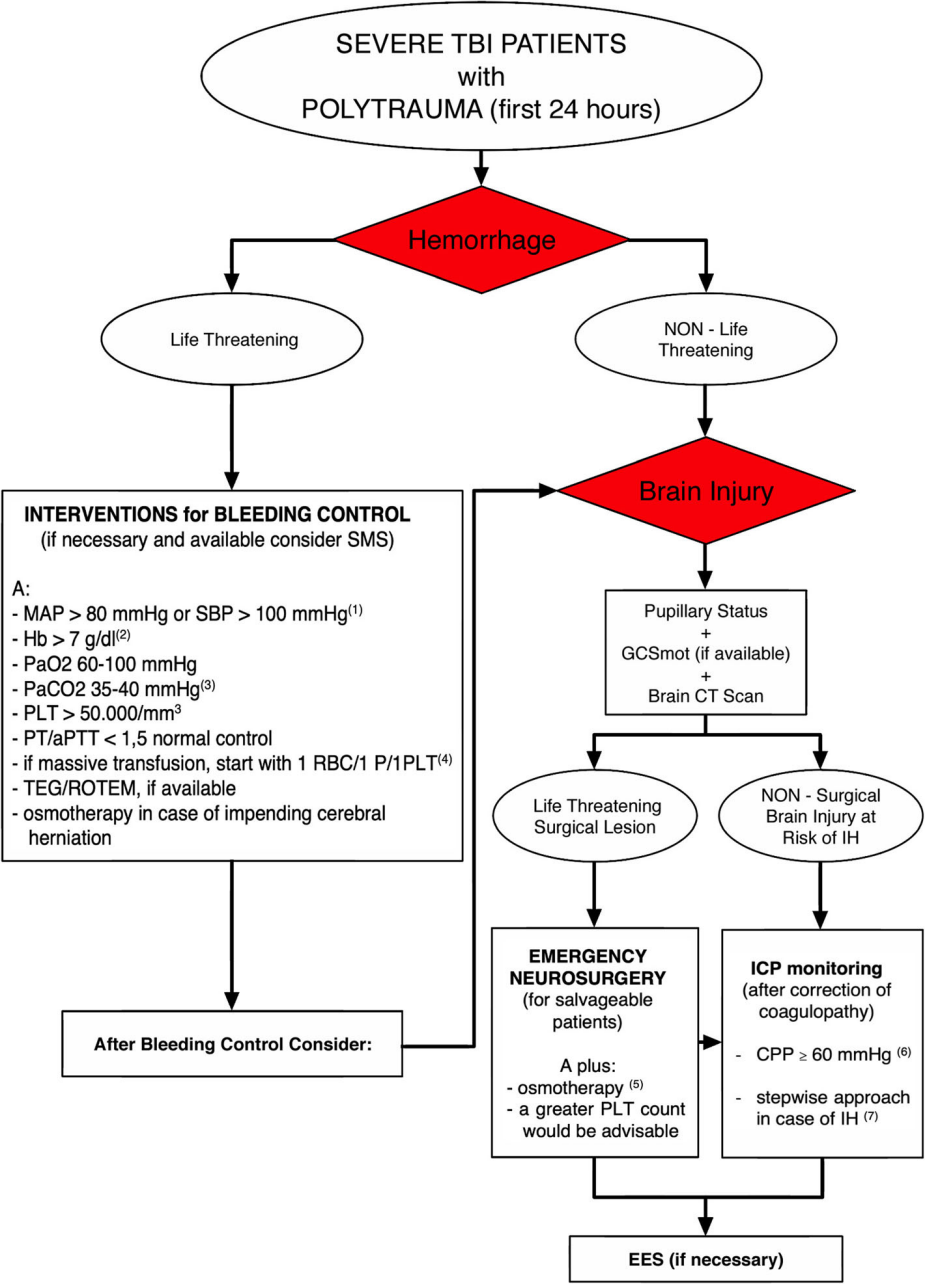
Consensus algorithm. (1) Lower values could be tolerated, for the shortest possible time, in case of difficult intraoperative bleeding control. (2) Higher threshold could be used in patients “at risk” (i.e., elderly and/or with limited cardiovascular reserve because of pre-existing heart disease). (3) Lower values, temporarily, only in case of impending cerebral herniation. (4) Afterwards, this ratio can be modified according to laboratory values. (5) Not only in case of impending cerebral herniation but also for cerebral edema control. (6) This value should be adjusted (individualized) considering neuromonitoring data and cerebral autoregulation status. (7) This approach is recommended in the absence of possibilities to target the underlying pathophysiologic mechanism of IH. *Abbreviations:* SMS = systemic multisystem surgery (including radiologic interventional procedures), CT = computed tomography, GCS = Glasgow Coma Scale (mot = motor part of GCS), MAP = mean arterial pressure, SBP = systolic blood pressure, Hb = hemoglobin, PaO2 = arterial partial pressure of oxygen, PaCO2 = arterial partial pressure of carbon dioxide, RBC = red blood cell, P = plasma, PLT = platelet, PT = prothrombin time, aPTT = activated partial thromboplastin time, TEG = thromboelastography, ROTEM = rotational thromboelastometry, ICP = intracranial pressure, CPP = cerebral perfusion pressure, IH = intracranial hypertension, EES extracranial emergency surgery

Agreement was reached on sixteen recommendations (Table 1); they are listed below with the percentage of agreement and associated comments. Figure 1 shows the consensus algorithm.

**Table 1:** Summary of consensus conference recommendations

Number	Recommendation	Agreement (%)
1	All exsanguinating patients (life-threatening hemorrhage) require immediate intervention (surgery and/or interventional radiology) for bleeding control.	100
2	Patients without life-threatening hemorrhage or following measures to obtain bleeding control (in case of life-threatening hemorrhage) require urgent neurological evaluation [pupils + Glasgow Coma Scale motor score (if feasible), and brain computed tomography (CT) scan] to determine the severity of brain damage (life-threatening or not).	100
3	After control of life-threatening hemorrhage is established, all salvageable patients with life-threatening brain lesions require urgent neurosurgical consultation and intervention.	100
4	Patients (without or after control of life-threatening hemorrhage) at risk for intracranial hypertension (IH)* (without a life-threatening intracranial mass lesion or after emergency neurosurgery) require intracranial pressure (ICP) monitoring regardless of the need of emergency extra-cranial surgery (EES) [16, 17].	97.5
5	We recommend maintaining systolic blood pressure (SBP) > 100 mmHg or mean arterial pressure (MAP) > 80 mmHg during interventions for life-threatening hemorrhage or emergency neurosurgery. In cases of difficult intraoperative bleeding control, lower value may be tolerated for the shortest possible time.	82.5
6	We recommend red blood cell (RBC) transfusion for hemoglobin (Hb) level < 7 g/dl during interventions for life-threatening hemorrhage or emergency neurosurgery. Higher threshold for RBC transfusions may be used in patients “at risk” (i.e., the elderly and/or patients with limited cardiovascular reserve due to pre-existing heart disease).	97.5
7	We recommend maintaining an arterial partial pressure of oxygen (PaO2) level between 60 and 100 mmHg during interventions for life-threatening hemorrhage or emergency neurosurgery.	95
8	We recommend maintaining an arterial partial pressure of carbon dioxide (PaCO2) level between 35 and 40 mmHg during interventions for life-threatening hemorrhage or emergency neurosurgery.	97.5
9	In cases of cerebral herniation, awaiting or during emergency neurosurgery, we recommend the use of osmotherapy and/or hypocapnia (temporarily).	90
10	In cases requiring intervention for life-threatening systemic hemorrhage, we recommend, at a minimum, the maintenance of a platelet (PLT) count > 50.000/mm^3^. In cases requiring emergency neurosurgery (including ICP probe insertion), a higher value is advisable.	100
11	We recommend maintaining a prothrombin time (PT)/activated partial thromboplastin time (aPTT) value of < 1.5 normal control during interventions for life-threatening hemorrhage or emergency neurosurgery (including ICP probe insertion).	92.5
12	We recommend, if available, that Point-of-Care (POC) tests [e.g., thromboelastography (TEG) and rotational thromboelastometry ROTEM] be utilized to assess and optimize coagulation function during interventions for life-threatening hemorrhage or emergency neurosurgery (including ICP probe insertion).	90
13	During massive transfusion protocol initiation, we recommend the transfusion of RBCs/plasma/PLTs at a ratio of 1/1/1. Afterwards, this ratio may be modified according to laboratory values.	92.5
14	We recommend maintaining a cerebral perfusion pressure (CPP) ≥ 60 mmHg when ICP monitoring becomes available. This value should be adjusted (individualized) based on neuromonitoring data and the cerebral autoregulation status of the individual patient.	95
15	In the absence of possibilities to target the underlying pathophysiologic mechanism of IH, we recommend a stepwise approach [18], where the level of therapy, in patients with elevated ICP, is increased step by step, reserving more aggressive interventions, which are generally associated with greater risks/adverse effects, for situations when no response is observed.	97.5
16	We recommend the development of protocols, in conjunction with local resources and practices, to encourage the implementation of a simultaneous multisystem surgery (SMS) [including radiologic interventional procedures] in patients requiring both intervention for life-threatening hemorrhage and emergency neurosurgery for life-threatening brain damage.	100

*Patients in coma with radiological signs of intracranial hypertension

### Recommendation 1

All exsanguinating patients (life-threatening hemorrhage) require immediate intervention (surgery and/or interventional radiology) for bleeding control.

Agreement: 100%.

### Recommendation 2

Patients without life-threatening hemorrhage or following measures to obtain bleeding control (in case of life-threatening hemorrhage) require urgent neurological evaluation [pupils + Glasgow Coma Scale (GCS) motor score (if feasible), and brain computed tomography (CT) scan] to determine the severity of brain damage (life-threatening or not).

Agreement: 100%.

### Recommendation 3

After control of life-threatening hemorrhage is established, all salvageable patients with life-threatening brain lesions require urgent neurosurgical consultation and intervention.

Agreement: 100%.

### Recommendation 4

Patients (without or after control of life-threatening hemorrhage) at risk for intracranial hypertension (IH)* (without a life-threatening intracranial mass lesion or after emergency neurosurgery) require intracranial pressure (ICP) monitoring regardless of the need of emergency extra-cranial surgery (EES) [16, 17].

* = patients in coma with radiological signs of IH.

*Agreement: 97.5%.*

### Recommendation 5

We recommend maintaining systolic blood pressure (SBP) > 100 mmHg or mean arterial pressure (MAP) > 80 mmHg during interventions for life-threatening hemorrhage or emergency neurosurgery. In cases of difficult intraoperative bleeding control, lower values may be tolerated for the shortest possible time.

Agreement: 82.5%.

### Recommendation 6

We recommend red blood cell (RBC) transfusion for hemoglobin (Hb) level < 7 g/dl during interventions for life-threatening hemorrhage or emergency neurosurgery. Higher threshold for RBC transfusions may be used in patients “at risk” (i.e. the elderly and/or patients with limited cardiovascular reserve due to pre-existing heart disease).

Agreement: 97.5 %.

### Recommendation 7

We recommend maintaining an arterial partial pressure of oxygen (PaO2) level between 60 and 100 mmHg during interventions for life-threatening hemorrhage or emergency neurosurgery.

Agreement: 95%.

### Recommendation 8

We recommend maintaining an arterial partial pressure of carbon dioxide (PaCO2) level between 35 and 40 mmHg during interventions for life-threatening hemorrhage or emergency neurosurgery.

Agreement: 97.5%.

### Recommendation 9

In cases of cerebral herniation, awaiting or during emergency neurosurgery, we recommend the use of osmotherapy and/or hypocapnia (temporarily).

Agreement: 90%.

### Recommendation 10

In cases requiring intervention for life-threatening systemic hemorrhage, we recommend, at a minimum, the maintenance of a platelet (PLT) count > 50.000/mm^3^. In cases requiring emergency neurosurgery (including ICP probe insertion), a higher value is advisable.

Agreement: 100%.

### Recommendation 11

We recommend maintaining a prothrombin time (PT)/activated partial thromboplastin time (aPTT) value of < 1.5 normal control during interventions for life-threatening hemorrhage or emergency neurosurgery (including ICP probe insertion).

Agreement: 92.5%.

### Recommendation 12

We recommend, if available, that point-of-care (POC) tests [e.g., thromboelastography (TEG) and rotational thromboelastometry ROTEM] be utilized to assess and optimize coagulation function during interventions for life-threatening hemorrhage or emergency neurosurgery (including ICP probe insertion).

Agreement: 90%.

### Recommendation 13

During massive transfusion protocol initiation, we recommend the transfusion of RBCs/Plasma/PLTs at a ratio of 1/1/1. Afterwards, this ratio may be modified according to laboratory values.

Agreement: 92.5%.

### Recommendation 14

We recommend maintaining a cerebral perfusion pressure (CPP) ≥ 60 mmHg when ICP monitoring becomes available. This value should be adjusted (individualized) based on neuromonitoring data and the cerebral autoregulation status of the individual patient.

Agreement: 95%.

### Recommendation 15

In the absence of possibilities to target the underlying pathophysiologic mechanism of IH, we recommend a stepwise approach [18], where the level of therapy, in patients with elevated ICP, is increased step by step, reserving more aggressive interventions, which are generally associated with greater risks/adverse effects, for situations when no response is observed.

Agreement: 97.5%.

### Recommendation 16

We recommend the development of protocols, in conjunction with local resources and practices, to encourage the implementation of a simultaneous multisystem surgery (SMS) [including radiologic interventional procedures] in patients requiring both intervention for life-threatening hemorrhage and emergency neurosurgery for life-threatening brain damage.

Agreement: 100%.

## Discussion

### Critical clinical decisions regarding hemorrhage control in TBI polytrauma patients

Life-threatening hemorrhage is one of the major preventable causes of early death after trauma [3–5]. Therefore, precise and early control of hemorrhage, with associated restoration of circulating blood volume, remains a priority [9, 19, 20]. It is well accepted that hemorrhage can be controlled by damage control surgery and/or interventional radiology [8, 21]. Typically, a basic clinical neurological evaluation (GCS motor score + pupils) with a brain CT scan is necessary both to determine the patient’s salvageability and to address the possible need for additional monitoring and urgent neurosurgical intervention [13, 19, 22]. Often, uncontrolled hemorrhage in TBI polytrauma patients may require simultaneous multisystem surgery [23–25]. The main objective should be the control of bleeding and the avoidance/minimization of secondary brain insults. This approach, frequently adopted in the war trauma setting, but rarely in the civilian one, requires established protocols and a strict collaboration between different surgical teams (including interventional radiologists) [23]. Kinoshita et al. performed a retrospective study to evaluate the efficacy of a hybrid emergency room (capable of deploying SMS) on functional outcomes in TBI polytrauma patients [24]. This system was significantly associated with both shorter times to initiate CT scanning/emergency surgery and fewer unfavorable outcomes at 6 months post-injury. The results of a recent survey [12] showed that, although few centers are currently equipped to perform SMS for hemorrhage in TBI polytrauma patients, the majority of the responding centers considered the ability to perform SMS as important, very important, or even mandatory. Although this consensus reinforces the implementation of this approach, future studies designed to evaluate the usefulness of SMS in polytrauma TBI patients are warranted.

### Preservation/protection of the injured brain during interventions for extra-cranial bleeding control

In TBI polytrauma patients, it is mandatory to minimize secondary or delayed insults, like hypoxia and arterial hypotension, while emergency surgeons control extra-cranial bleeding. Hypotension (defined as a SBP < 90 mmHg) is a well-recognized secondary insult, known to be associated with unfavorable neurological outcome [26, 27]. Moreover, recent observational studies suggest that the currently established threshold of 90 mmHg may, in fact, be too low [28, 29]. Further trials are required to identify the correct SBP value in this setting. While Brain Trauma Foundation (BTF) guidelines suggest that SBP be maintained at ≥ 100 mmHg for patients 50–69 years or at a minimum of ≥ 110 mmHg for patients 15–49 years or older than 70 years [13], we have chosen a value of 100 mmHg as a threshold for bleeding TBI polytrauma patients. Furthermore, we suggest that lower values of SBP be maintained for the shortest possible time, particularly in cases associated with difficult intraoperative bleeding control.

The optimal Hb value in TBI polytrauma patients remains to be determined. The Transfusion Requirements in Critical Care (TRICC) study showed no differences in 30-day mortality between the use of a liberal transfusion strategy (trigger for transfusion Hb > 10 g/dl) and the use of a more restrictive transfusion strategy (trigger for transfusion Hb > 7 g/dl) in 838 critically ill patients [30]. A subgroup analysis of the TRICC trial, focusing on 67 severe TBI patients, confirmed no survival benefit comparing the liberal vs. the restrictive transfusion strategy [31]. Robertson et al. [32] reported the results of a randomized clinical trial designed to compare the effects of erythropoietin and two hemoglobin transfusion thresholds (7 and 10 g/dL) on neurological recovery after TBI. These investigators found that the administration of erythropoietin or the maintenance of Hb value > 10 g/dL was not associated with improved neurological outcome at 6 months. Moreover, the use of a transfusion threshold of 10 g/dL was associated with a higher incidence of adverse events. Given the absence of additional published studies, we recommend a Hb threshold of 7 g/dl in TBI polytrauma patients. Higher thresholds for RBCs transfusions in patients “at risk” (i.e., elderly and/or with limited cardiovascular reserve because of pre-existing heart disease) may be considered [30].

Randomized controlled trials targeting the optimal PaO2 and PaCO2 values in TBI polytrauma patients are lacking. The presence of hypoxia, historically and pathophysiologically defined as a peripheral oxygen saturation (SpO2) < 90% (corresponding near to a PaO2 of 60 mmHg), has been associated with poor outcomes in TBI patients both in the pre-hospital and in-hospital setting [27, 33, 34]. A retrospective study, enrolling 3420 severe TBI patients, showed that both a PaO2 < 110 mmHg and a PaO2 > 487 mmHg were associated with increased mortality and worsened neurological outcomes [35]. Another retrospective study, involving 1547 severe TBI patients, reported (1) an association between early (within 24 hours from admission) hyperoxia (defined as a PaO2 > 200 mmHg) and mortality/short-term functional outcomes (lower GCS discharge scores), and (2) an association between a PaO2 < 100 mmHg and mortality [36]. The authors suggest that the negative effects of hyperoxia may have been related to hyperoxia-induced oxygen-free radical toxicity. However, a transient hyperoxia, achieved by increasing the oxygen content and delivery, may be potentially beneficial in trauma patients with severe anemia [37]. Hypocapnia, induced by hyperventilation, is also known to be associated with the risk of development of cerebral ischemia [38] and worsened neurological outcome after TBI [39]. Moreover, in cases of hypovolemia, an increase in airway pressure (sometimes associated with hyperventilation) can reduce venous return, thereby inducing or exacerbating arterial hypotension [40].

Platelets are known to play a key role in hemostasis after trauma [41]. A reduction in PLT count is associated with an increase in mortality and the progression of post-traumatic intracranial bleeding [42–44]. Recent guidelines recommend the maintenance of a PLT count > 50.000/mm^3^ (grade 1 C) in polytrauma patients and further recommend a more stringent cut-off (> 100.000/mm^3^) in case of ongoing bleeding and/or TBI (grade 2 C) [10]. Furthermore, coagulopathy is frequently observed after trauma and is often associated with increased mortality [41, 45]. In TBI polytrauma patients, coagulopathy is associated with intracranial bleeding progression and unfavorable neurological outcomes [46, 47].

Massive transfusion is frequently utilized in trauma patients [19, 20]. The Pragmatic Randomized Optimal Platelet and Plasma Ratios (PROPPR) study, involving 680 trauma patients with major bleeding, was performed to determine the safety and the effectiveness of a transfusion strategy involving plasma, PLTs, and RBCs in a 1:1:1 ratio compared with a 1:1:2 ratio. This study showed that none of the strategies resulted in significant differences in mortality. However, more patients in the 1:1:1 group achieved hemostasis and fewer experienced death due to exsanguination within the first 24 hours [48]. Given the negative effects of coagulopathy on TBI (42–44, 46–47), we recommend the initiation of a transfusion protocol of RBCs/plasma/PLTs at a ratio of 1:1:1. This ratio may be modified afterwards according to laboratory values.

Point-of-care tests (i.e., TEG, ROTEM, etc.) are increasingly used in the evaluation of coagulation function in trauma patients with hemorrhagic complications [10, 20, 41]. These tests can be utilized to obtain a rapid assessment of hemostasis and to assist in clinical decision-making; they can further provide critical information about specific coagulation deficiencies [10, 41, 49]. Moreover, they can be particularly useful in patients taking novel oral anticoagulants (NOACs) and in the evaluation of PLT dysfunction induced by trauma and/or drugs [10]. In light of the above, these tests may be useful in TBI polytrauma patients [50].

## Conclusions

Future studies are needed and should be encouraged to improve clinical outcomes in this challenging setting. In the absence of more compelling data, the present practical consensus conference was intended to establish and provide a shared, multidisciplinary approach to deliver the best possible care during the very early stages of management of TBI polytrauma patients.

## Supplementary information

**Additional file 1.** Appendix 1. List of participants.

## Data Availability

The datasets used and/or analyzed during the current study are available from the corresponding author on reasonable request.

## Abbreviations

AIS: Abbreviated Injury Score
aPTT: Activated partial thromboplastin time
BTF: Brain Trauma Foundation
CPP: Cerebral perfusion pressure
CT: Computed tomography
EES: Emergency extra-cranial surgery
GCS: Glasgow Coma Scale
Hb: Hemoglobin
ICP: Intracranial pressure
IH: Intracranial hypertension
MAP: Mean arterial pressure
NOACs: Novel oral anticoagulants
PaCO2: Arterial partial pressure of carbon dioxide
PaO2: Arterial partial pressure of oxygen
PLT: Platelet
POC: Point-of-care
PROPPR: Pragmatic Randomized Optimal Platelet and Plasma Ratios
PT: Prothrombin time
RBC: Red blood cell
ROTEM: Rotational thromboelastometry
SBP: Systolic blood pressure
SMS: Simultaneous multisystem surgery
SpO2: Peripheral oxygen saturation
TBI: Traumatic brain injury
TEG: Thromboelastography
TRICC: Transfusion Requirements in Critical Care
WSES: World Society of Emergency Surgery

## References

[1] Maas AIR, Menon DK, Adelson PD, Andelic N, Bell MJ, Belli A (2017). InTBIR Participants and Investigators. Traumatic brain injury: integrated approaches to improve prevention, clinical care, and research. Lancet Neurol.

[2] Dewan MC, Rattani A, Gupta S, Baticulon RE, Hung YC, Punchak M (2019). Estimating the global incidence of traumatic brain injury. J Neurosurg.

[3] Teixeira PG, Inaba K, Hadjizacharia P, Brown C, Salim A, Rhee P (2007). Preventable or potentially preventable mortality at a mature trauma center. J Trauma.

[4] Lozano R, Naghavi M, Foreman K, Lim S, Shibuya K, Aboyans V (2012). Global and regional mortality from 235 causes of death for 20 age groups in 1990 and 2010: a systematic analysis for the Global Burden of Disease Study2010. Lancet..

[5] Callcut RA, Kornblith LZ, Conroy AS, Robles AJ, Meizoso JP, Namias N (2019). Western Trauma Association Multicenter Study Group. The why and how our trauma patients die: a prospective Multicenter Western Trauma Association study. J Trauma Acute Care Surg.

[6] Dutton RP, Mackenzie CF, Scalea TM (2002). Hypotensive resuscitation during active hemorrhage: impact on in-hospital mortality. J Trauma.

[7] Jansen JO, Thomas R, Loudon MA, Brooks A (2009). Damage control resuscitation for patients with major trauma. BMJ.

[8] Gruen RL, Brohi K, Schreiber M, Balogh ZJ, Pitt V, Narayan M (2012). Haemorrhage control in severely injured patients. Lancet..

[9] Langan NR, Eckert M, Martin MJ (2014). Changing patterns of in-hospital deaths following implementation of damage control resuscitation practices in US forward military treatment facilities. JAMA Surg.

[10] Spahn DR, Bouillon B, Cerny V, Duranteau J, Filipescu D, Hunt BJ (2019). The European guideline on management of major bleeding and coagulopathy following trauma: fifth edition. Crit Care.

[11] Galvagno SM, Fox EE, Appana SN, Baraniuk S, Bosarge PL, Bulger EM (2017). PROPPR Study Group. Outcomes after concomitant traumatic brain injury and hemorrhagic shock: a secondary analysis from the Pragmatic, Randomized Optimal Platelets and Plasma Ratios trial. J Trauma Acute Care Surg.

[12] Picetti E, Maier RV, Rossi S, Kirkpatrick AW, Biffl WL, Stahel PF (2019). Preserve encephalus in surgery of trauma: online survey. (P.E.S.T.O). World J Emerg Surg.

[13] Carney N, Totten AM, O'Reilly C, Ullman JS, Hawryluk GW, Bell MJ (2017). Guidelines for the management of severe traumatic brain injury, fourth edition. Neurosurgery..

[14] Cariou A, Payen JF, Asehnoune K, Audibert G, Botte A, Brissaud O (2017). Société de Réanimation de Langue Française (SRLF) and the Société Française d’Anesthésie et de Réanimation (SFAR) In conjunction with the Association de Neuro Anesthésie Réanimation de Langue Française (ANARLF), the Groupe Francophone de Réanimation et Urgences Pédiatriques (GFRUP), the Société Française de Médecine d’Urgence (SFMU), and the Société Française Neuro-Vasculaire (SFNV). Targeted temperature management in the ICU: guidelines from a French expert panel. Ann Intensive Care.

[15] Andrews PJD, Verma V, Healy M, Lavinio A, Curtis C, Reddy U (2018). Targeted temperature management in patients with intracerebral haemorrhage, subarachnoid haemorrhage, or acute ischaemic stroke: consensus recommendations. Br J Anaesth.

[16] Stocchetti N, Picetti E, Berardino M, Buki A, Chesnut RM, Fountas KN (2014). Clinical applications of intracranial pressure monitoring in traumatic brain injury: report of the Milan consensus conference. Acta Neurochir.

[17] Chesnut Randall, Videtta Walter, Vespa Paul, Le Roux Peter (2014). Intracranial Pressure Monitoring: Fundamental Considerations and Rationale for Monitoring. Neurocritical Care.

[18] Stocchetti N, Maas AI (2014). Traumatic intracranial hypertension. N Engl J Med.

[19] American College of Surgeons. Advanced Trauma Life Support® Student Course Manual. Thenth edition. 2018.

[20] Cannon JW (2018). Hemorrhagic shock. N Engl J Med.

[21] Benz D, Balogh ZJ (2017). Damage control surgery: current state and future directions. Curr Opin Crit Care.

[22] Bullock MR, Chesnut R, Ghajar J, Gordon D, Hartl R, Newell DW (2006). Surgical Management of Traumatic Brain Injury Author Group. Guidelines for the surgical management of traumatic brain injury. Neurosurgery..

[23] Moore JM, Thomas PA, Gruen RL, Chan P, Rosenfeld JV (2016). Simultaneous multisystem surgery: an important capability for the civilian trauma hospital. Clin Neurol Neurosurg.

[24] Kinoshita T, Hayashi M, Yamakawa K, Watanabe A, Yoshimura J, Hamasaki T (2018). Effect of the hybrid emergency room system on functional outcome in patients with severe traumatic brain injury. World Neurosurg.

[25] Carver D, Kirkpatrick AW, D'Amours S, Hameed SM, Beveridge J, Ball CG. A prospective evaluation of the utility of a hybrid operating suite for severely injured patients: overstated or underutilized ? Ann Surg. 2018; Dec 20. [Epub ahead of print]. 10.1097/SLA.0000000000003175 30601253

[26] Marmarou A, Anderson RL, Ward JD, Choi SC, Young HF (1991). Impact of ICP instability and hypotension on outcome in patients with severe head trauma. J Neurosurg.

[27] Chesnut RM, Marshall LF, Klauber MR, Blunt BA, Baldwin N, Eisenberg HM (1993). The role of secondary brain injury in determining outcome from severe head injury. J Trauma.

[28] Brenner M, Stein DM, Hu PF, Aarabi B, Sheth K, Scalea TM (2012). Traditional systolic blood pressure targets underestimate hypotension-induced secondary brain injury. J Trauma Acute Care Surg.

[29] Spaite DW, Hu C, Bobrow BJ, Chikani V, Sherrill D, Barnhart B (2017). Mortality and prehospital blood pressure in patients with major traumatic brain injury: implications for the hypotension threshold. JAMA Surg.

[30] Hébert PC, Wells G, Blajchman MA, Marshall J, Martin C, Pagliarello G (1999). A multicenter, randomized, controlled clinical trial of transfusion requirements in critical care. Transfusion Requirements in Critical Care Investigators, Canadian Critical Care Trials Group. N Engl J Med.

[31] McIntyre LA, Fergusson DA, Hutchison JS, Pagliarello G, Marshall JC, Yetisir E (2006). Effect of a liberal versus restrictive transfusion strategy on mortality in patients with moderate to severe head injury. Neurocrit Care.

[32] Robertson CS, Hannay HJ, Yamal JM, Gopinath S, Goodman JC, Tilley BC (2014). Epo Severe TBI Trial Investigators. Effect of erythropoietin and transfusion threshold on neurological recovery after traumatic brain injury: a randomized clinical trial. JAMA..

[33] Stocchetti N, Furlan A, Volta F (1996). Hypoxemia and arterial hypotension at the accident scene in head injury. J Trauma.

[34] Chi JH, Knudson MM, Vassar MJ, McCarthy MC, Shapiro MB, Mallet S (2006). Prehospital hypoxia affects outcome in patients with traumatic brain injury: a prospective multicenter study. J Trauma.

[35] Davis DP, Meade W, Sise MJ, Kennedy F, Simon F, Tominaga G (2009). Both hypoxemia and extreme hyperoxemia may be detrimental in patients with severe traumatic brain injury. J Neurotrauma.

[36] Brenner M, Stein D, Hu P, Kufera J, Wooford M, Scalea T (2012). Association between early hyperoxia and worse outcomes after traumatic brain injury. Arch Surg.

[37] Vincent JL, Taccone FS, He X (2017). Harmful effects of hyperoxia in postcardiac arrest, sepsis, traumatic brain injury, or stroke: the importance of individualized oxygen therapy in critically ill patients. Can Respir J.

[38] Coles JP, Fryer TD, Coleman MR, Smielewski P, Gupta AK, Minhas PS (2007). Hyperventilation following head injury: effect on ischemic burden and cerebral oxidative metabolism. Crit Care Med.

[39] Muizelaar JP, Marmarou A, Ward JD, Kontos HA, Choi SC, Becker DP (1991). Adverse effects of prolonged hyperventilation in patients with severe head injury: a randomized clinical trial. J Neurosurg.

[40] Davis DP (2008). Early ventilation in traumatic brain injury. Resuscitation..

[41] Kornblith LZ, Moore HB, Cohen MJ (2019). Trauma-induced coagulopathy: The past, present, and future. J Thromb Haemost.

[42] Stansbury LG, Hess AS, Thompson K, Kramer B, Scalea TM, Hess JR (2013). The clinical significance of platelet counts in the first 24 hours after severe injury. Transfusion..

[43] Schnuriger B, Inaba K, Abdelsayed GA, Lustenberger T, Eberle BM, Barmparas G (2010). The impact of platelets on the progression of traumatic intracranial hemorrhage. J Trauma.

[44] Joseph B, Pandit V, Meyer D, Butvidas L, Kulvatunyou N, Khalil M (2014). The significance of platelet count in traumatic brain injury patients on antiplatelet therapy. J Trauma Acute Care Surg.

[45] MacLeod JB, Lynn M, McKenney MG, Cohn SM, Murtha M (2003). Early coagulopathy predicts mortality in trauma. J Trauma.

[46] Allard CB, Scarpelini S, Rhind SG, Baker AJ, Shek PN, Tien H (2009). Abnormal coagulation tests are associated with progression of traumatic intracranial hemorrhage. J Trauma.

[47] Yuan Q, Sun YR, Wu X, Yu J, Li ZQ, Du ZY (2016). Coagulopathy in traumatic brain injury and its correlation with progressive hemorrhagic injury: a systematic review and meta-analysis. J Neurotrauma.

[48] Holcomb JB, Tilley BC, Baraniuk S, Fox EE, Wade CE, Podbielski JM (2015). PROPPR Study Group. Transfusion of plasma, platelets, and red blood cells in a 1:1:1 vs a 1:1:2 ratio and mortality in patients with severe trauma: the PROPPR randomized clinical trial. JAMA..

[49] Moore Ernest E., Moore Hunter B., Chapman Michael P., Gonzalez Eduardo, Sauaia Angela (2018). Goal-directed hemostatic resuscitation for trauma induced coagulopathy. Journal of Trauma and Acute Care Surgery.

[50] Kvint S, Schuster J, Kumar MA (2017). Neurosurgical applications of viscoelastic hemostatic assays. Neurosurg Focus.

