# Supplementary material for: WSES consensus conference guidelines: monitoring and management of severe adult traumatic brain injury patients with polytrauma in the first 24 hours
Source: World J Emerg Surg. 2019 Nov 29;14:53. doi: 10.1186/s13017-019-0270-1 (PMC6884766; doi:10.1186/s13017-019-0270-1)
Supplement: Supplementary file 1 — Additional file 1. Appendix 1. List of participants. [file 13017_2019_270_MOESM1_ESM.docx]

1. Fikri M Abu-Zidan (United Arab Emirates)
2. Luca Ansaloni (Italy)
3. Rocco Armonda (USA)
4. Miklosh Bala (Israel)
5. Zsolt J Balogh (Australia)
6. Maurizio Berardino (Italy)
7. Walter L Biffl (USA)
8. Pierre Bouzat (France)
9. Andras Buki (Hungary)
10. Fausto Catena (Italy)
11. Randal M Chesnut (USA)
12. Osvaldo Chiara (Italy)
13. Giuseppe Citerio (Italy)
14. Federico Coccolini (Italy)
15. Raul Coimbra (USA)
16. Salomone Di Saverio (UK)
17. Gustavo P Fraga (Brazil)
18. Deepak Gupta (India)
19. Raimund Helbok (Austria)
20. Peter J Hutchinson (UK)
21. Andrew W Kirkpatrick (Canada)
22. Takahiro Kinoshita (Japan)
23. Ari Leppaniemi (Finland)
24. Andrew IR Maas (Belgium)
25. Ronald V Maier (USA)
26. John A Myburgh (Australia)
27. David O Okonkwo (USA)
28. Yasuhiro Otomo (Japan)
29. Edoardo Picetti (Italy)
30. Sandro Rizoli (Canada)
31. Sandra Rossi (Italy)
32. Andres M Rubiano (Colombia)
33. Juan Sahuquillo (Spain)
34. Thomas M Scalea (USA)
35. Franco Servadei (ITA)
36. Philip F Stahel (USA)
37. Nino Stocchetti (Italy)
38. Fabio S Taccone (Belgium)
39. George Velmahos (USA)
40. Dieter Weber (Australia)
